# Mineral dust as a driver of carbon accumulation in northern latitudes

**DOI:** 10.1038/s41598-018-25162-9

**Published:** 2018-05-02

**Authors:** Malin E. Kylander, A. Martínez-Cortizas, Richard Bindler, Joeri Kaal, Jenny K. Sjöström, Sophia V. Hansson, Noemí Silva-Sánchez, Sarah L. Greenwood, Kerry Gallagher, Johan Rydberg, Carl-Magnus Mörth, Sebastien Rauch

**Affiliations:** 1 Department of Geological Sciences, Stockholm University, SE-10691 Stockholm, Sweden; 2 The Bolin Centre for Climate Research, Stockholm University, SE-10691 Stockholm, Sweden; 3 Departamento de Edafoloxía e Química Agrícola, Facultade de Bioloxía, Universidade de Santiago de Compostela, Campus Sur, E-15706 Santiago de Compostela, Spain; 4 Department of Ecology and Environmental Sciences, Umeå University, SE-901 87 Umeå, Sweden; 5 Department of Bioscience - Arctic Research Centre, Aarhus University, Frederiksborgvej 399, DK-4000 Roskilde, Denmark; 6 Géosciences, Université de Rennes 1, Rennes, 35042 France; 7 Water Environment Technology, Chalmers University of Technology, 41296 Göteborg, Sweden

## Abstract

Peatlands in northern latitudes sequester one third of the world’s soil organic carbon. Mineral dusts can affect the primary productivity of terrestrial systems through nutrient transport but this process has not yet been documented in these peat-rich regions. Here we analysed organic and inorganic fractions of an 8900-year-old sequence from Store Mosse (the “Great Bog”) in southern Sweden. Between 5420 and 4550 cal yr BP, we observe a seven-fold increase in net peat-accumulation rates corresponding to a maximum carbon-burial rate of 150 g C m^−2^ yr^−1^ – more than six times the global average. This high peat accumulation event occurs in parallel with a distinct change in the character of the dust deposited on the bog, which moves from being dominated by clay minerals to less weathered, phosphate and feldspar minerals. We hypothesize that this shift boosted nutrient input to the bog and stimulated ecosystem productivity. This study shows that diffuse sources and dust dynamics in northern temperate latitudes, often overlooked by the dust community in favour of arid and semi-arid regions, can be important drivers of peatland carbon accumulation and by extension, global climate, warranting further consideration in predictions of future climate variability.

## Introduction

In previously glaciated regions of the mid- and high-latitudes climatic conditions are often wet and cold, providing optimal environments for peat accumulation, where primary productivity exceeds ecosystem respiration. Northern boreal and sub-Arctic peatlands are estimated to store one third of the global soil organic carbon^1^ and could release up to 100 Mt of carbon per year per 1 °C increase as our climate continues to warm^2,3^. Compilations of global carbon stocks in peatlands necessarily require simplifications, such as the assumptions of constant deposit thicknesses and net peat accumulation rates (PAR). However, PAR, and hence net carbon burial, can vary widely over time, through the complex interplay between a number of climate mediated factors including deposit hydrology, surface vegetation and nutrient availability^1^. In their capacity as a nutrient vehicle mineral dusts have the potential to impact nutrient-poor systems and their primary productivity^4^, but given the rapid nature of carbon cycling in many ecosystems, this effect is hard to quantify. In bogs, decomposition processes slow considerably once buried organic material reaches the anoxic catotelm. This provides us with a window into the potential impacts of dust deposition on peat and carbon accumulation in a terrestrial ecosystem^5^.

As peatlands develop over time their upper layers can be increasingly isolated from the local hydrology and the surface vegetation eventually becomes reliant on atmospheric deposition alone for nutrient supply. Such ombrotrophic peatlands are considered to be among the most nutrient-poor ecosystems in the world^6^. Generally, these are phosphorus (P) limited^7,8^ and in some cases nitrogen:phosphorus (N:P) co-limited^9^. Although less studied, potassium (K) limitation also occurs^10^. Nutrient availability is thus a factor controlling bog productivity and by extension, carbon burial.

Since dust may contain significant amounts of e.g., K, P, Fe, Ca, S, Mg, it is an important agent of nutrient transport^11^. The nutrient balance of terrestrial ecosystems can be positively affected by dust deposition^4^ even at large spatial scales like that seen in the cross-continental fertilization of the Amazon rainforest by P and Fe-rich dust from the Bodélé depression^12^. Peat-rich temperate latitudes are often overlooked in terms of dust dynamics because emissions from these regions are not as spatially widespread as from lower latitude, arid regions and sources are often diffuse and difficult to pinpoint (soils, exposed bedrock, sand sheets, lake beds, dune systems). Even in the humid settings of temperate latitudes, however, dust emissions do occur, particularly in previously glaciated landscapes where there is an abundance of unconsolidated glacial material^13^. These glacigenic deposits have the potential to affect surrounding ecosystems and their productivity^14,15^.

**Figure 1:**
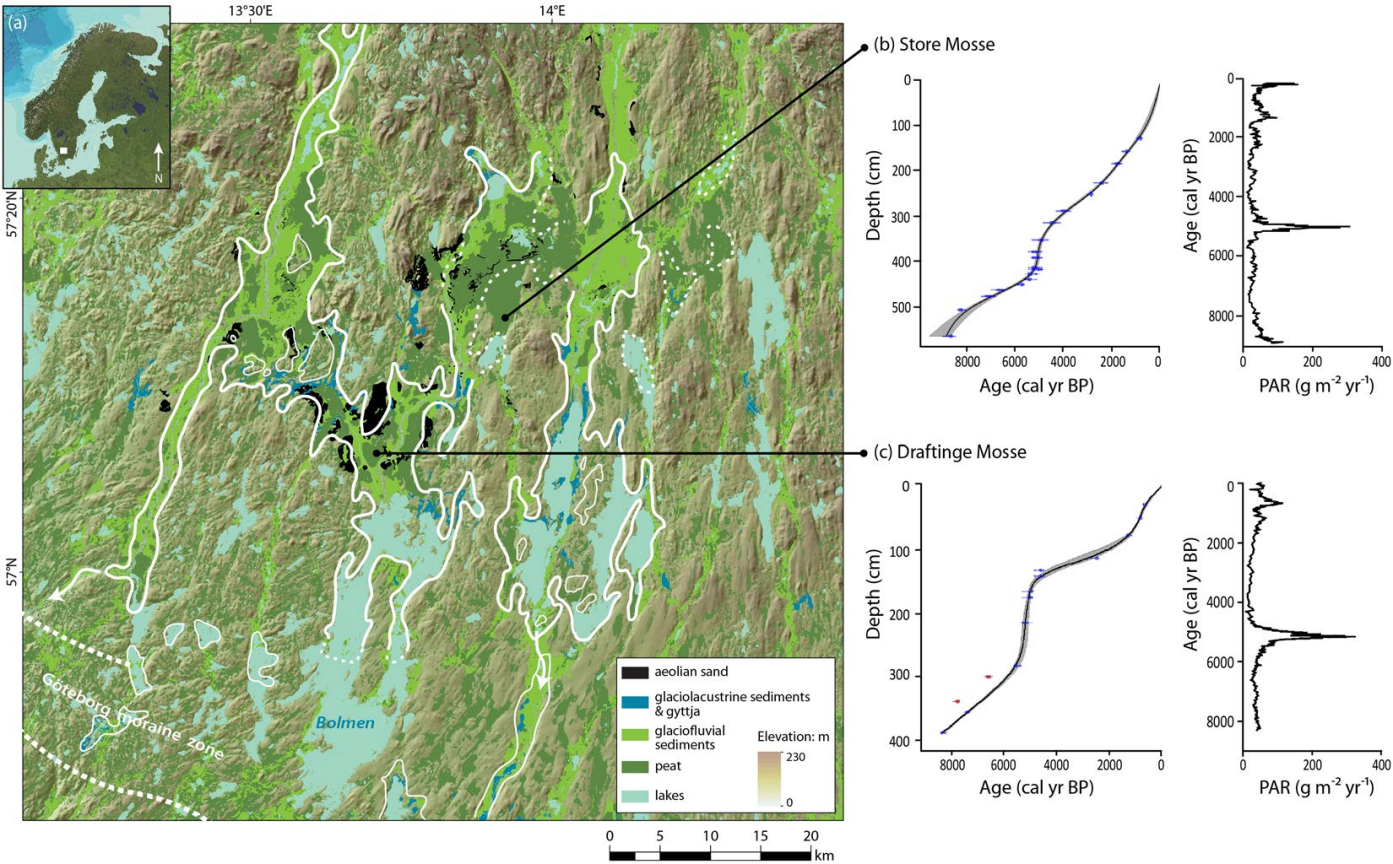
The study area in southern Sweden. This area was once covered by Fornbolmen, a large glacial ice lake (or lakes)(modelled boundaries of the main lakes shown by the white lines, with solid lines representing a greater degree of confidence than the dashed lines) (**a**). Draining of this lake due to isostatic uplift saw the exposed lake bottom being eroded by wind, leaving a large number of dunes and sand sheets in the area. The former lake bottom also made an ideal substrate for peat formation and today bogs in the area cover some 287 km^2^. According to the age model from this study (**b**) as well as previous work^17,23^, peat formation at Store Mosse began ~9000 years ago. What is unique is the HPAE observed between 5420 to 4550 years ago. An HPAE of similar timing and magnitude is found 18 km to the southwest at another bog, Draftinge Mosse (**c**). Note that the outliers in this latter age model are, in contrast to the majority of the samples, from bulk peat analyses.

**Figure 2:**
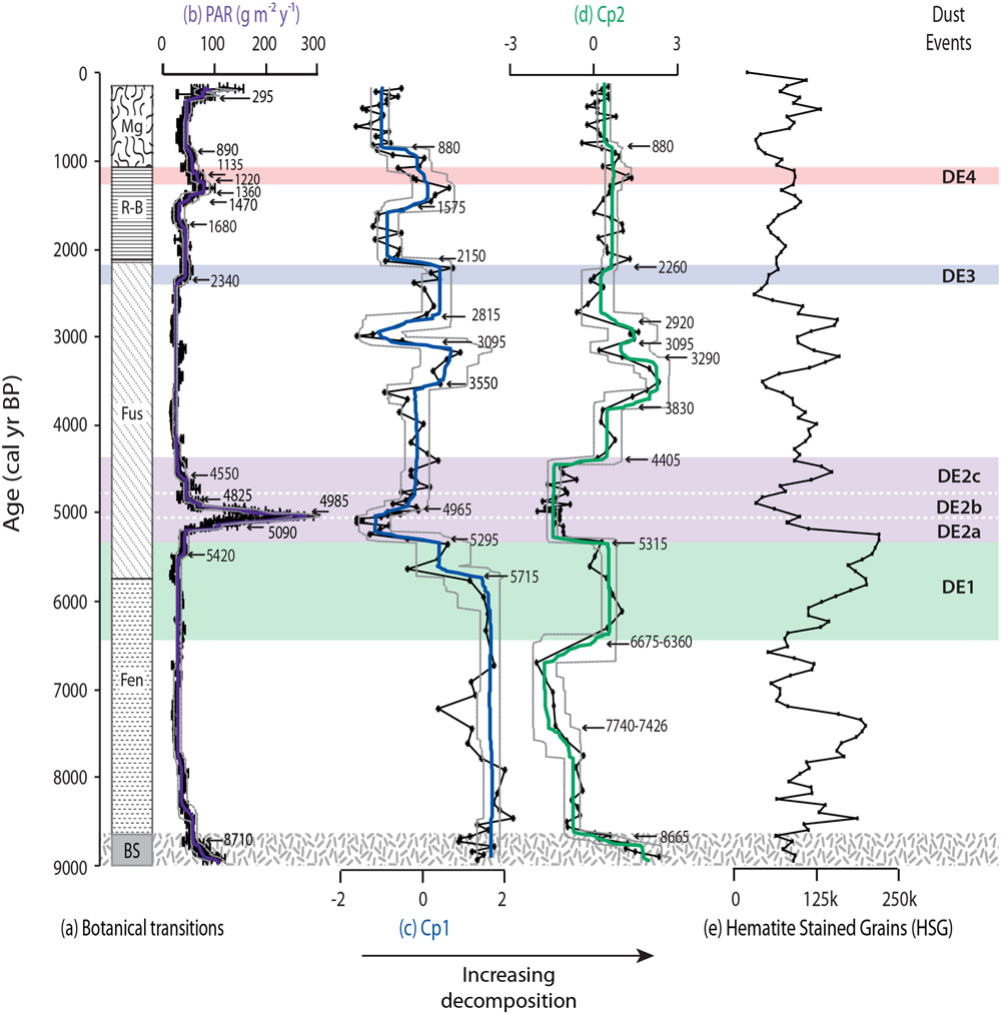
Peat Accumulation Rates (PAR), Principal Components (PC) based on OM proxies and Bond Events. The stage development (**a**) and the PAR (**b**) at Store Mosse are typical of ombrotrophic bogs with the exception of the PAR increase during the HPAE from 5420 to 4550 cal yr BP. A PCA made on the organic chemistry data, which includes total C and N, C/N, carbohydrates/lignin and carbohydrate/aliphatic ratios, δ^15^N, δ^13^C, and DPH, suggests that decomposition was low and that a higher abundance of the easily degraded compounds were preserved during the HPAE (**c**,**d**). This may be partially in response to the rewetting of the ecosystem that occurred with the end of the dry and cold Bond Event 4 (**e**). Errors on the PAR are the quartiles (25 to 75% of the data) from Monte Carlo simulations (see Methods). The solid coloured lines are the expected or mean value of the inferred function over time for each data type and the grey dashed lines are the 95% credible intervals from changepoint modelling.

We assess the links between terrestrial ecosystem productivity and dust deposition in a northern temperate setting using a peat sequence covering the last 8,900 years from Store Mosse (the “Great Bog”)(N57°13′37′′, E13°55′17′′), an extensive (77 km^2^) bog located in southern Sweden (Fig. 1a). Decades of research at this protected national park has revealed a uniform stratigraphy and temporally consistent botanical transitions from fen to *Sphagnum fuscum* to *S. rubellum/S. fuscum* and *S. magellanicum* stages^16,17^ (Fig. 2a). More recent studies reconstructing past atmospheric deposition based on dust-related elements (Al, Sc, Ti, Rb, U, Th, Y, and Rare Earth Elements (REE)) from total acid digestions and ICP-MS/AES analyses revealed atmospheric control of the site over the last 8,750 years. Four dust events (DE) were defined: DE1, DE3 and DE4 are periods of increased dust deposition, while DE2 shows a distinct source shift^18,19^. Here we present indicators of organic matter (OM) quantity (total C and N) and quality (C/N, carbohydrates/lignin and carbohydrate/aliphatic ratios, δ^15^N, δ^13^C, degree of humification (DPH)) from elemental, isotopic and Fourier transform infrared spectroscopy-Attenuated total reflectance (FTIR-ATR) analyses and relate this to dust deposition rates (as estimated based on Al mass accumulation rates (Al MAR)) and changes in dust source (Ga/Al, La/Lu, Gd/Lu, [Eu/Eu*]_UCC_). We also use pyrolysis-gas chromatography-mass spectrometry (Py-GC-MS) to further examine a selected portion of the record using organic molecular proxies. The age-depth model for the record is based on 19 macrofossil ^14^C dates modelled using CLAM v.2.2^20^ (Fig. 1b). Furthermore, we use Principal Component Analysis (PCA) and changepoint modelling in order to identify statistically significant changes in the records^21^.

## Results and Discussion

Store Mosse is typical of *Sphagnum*-dominated bogs in terms of the observed botanical transitions, bulk densities, OM content, C and N contents and C/N ratios^22^ (Fig. 2). PAR fluctuate around an average value of 45 g m^−2^ y^−1^, with slight increases at stage transitions (Fig. 2b). The average C content of the peat at Store Mosse is 49.0 ± 6.4% (2σ, *n* = 106) and therefore the observed C accumulation rates (CAR) at this site are in line with recent estimates of the global average time-weighted long-term CAR (22.9 ± 2.0 g C m^−2^ y^−1^)^22^. Starting 5420 cal yr BP however, a seven-fold increase in PAR occurs reaching a maximum value just 180 years later. This high peat accumulation event (HPAE) lasts just ~900 years as a gradual return to average background PAR is completed by 4550 cal yr BP. The maximum CAR during the HPAE (150 g C m^−2^ yr^−1^ at 5020 cal yr BP) is about six times higher than that normally observed in ombrotrophic peatlands worldwide and five times higher than Holocene maxima reported in a recent synthesis of northern peatlands^22^, making it truly remarkable. This distinct pattern of peat accumulation is confirmed by another core with comparable dating resolution from Store Mosse^23^. Convincingly, we have also found a mid-Holocene HPAE at Draftinge Mosse, a raised bog located 18 km to the southwest of Store Mosse, which is nearly identical in both timing and maximum CAR (156 g C m^−2^ yr^−1^ at 5170 cal yr BP) (Fig. 1c).

A PCA of the OM proxies at Store Mosse extracted two main components explaining 81% of the variance (Table 1). The first component, Cp1, is characterized by large positive loadings of C, δ^15^N, N and DPH, large negative loadings of carbohydrate/aliphatic and carbohydrate/lignin ratios, and moderate negative loadings of the C/N and δ^13^C ratios. C/N ratios and degree of peat humification from UV absorption of peat alkaline extracts are standard procedures used to determine the degree of peat decomposition^5,24^. Polysaccharides are more amenable to biological degradation than aliphatics or lignin, thus the ratio between these compounds changes with degree of decomposition^25–27^. At the same time, the δ^13^C ratio of the easily degradable components (i.e., polysaccharides, −22 to −24‰) is usually higher than that of more recalcitrant OM components (i.e., aliphatic and aromatic, −27 to −36‰)^28,29^ and peat C-isotopic composition becomes lighter (^13^C depleted) as the plant remains are degraded^30^, while δ^15^N increases with peat OM decomposition have been observed in various peat investigations^31,32^. Based on these indicators Cp1 represents the gradual decomposition of peat in the anoxic catotelm. Indeed, Cp1 scores are significantly correlated with the age of the peat (*n* = 105; r = 0.80; *P* < 0.001)(Fig. 2c) and higher Cp1 values are found in the lower sections of the profile (8900 until 5715 cal yr BP) indicating a higher degree of OM decomposition typical of basal sediments and fen peat. At the start of the *S. fuscum* stage decomposition decreases until 5295 cal yr BP and then drops rapidly during the HPAE. After that, values progressively increase until 4370 cal yr BP. Other relative increases in decomposition are found at 3550–3095, 2815–2150 and 1575–880 cal yr BP.

**Table 1:** PCA results from OM at Store Mosse.

Variable	Cp1	Cp2
C	0,91	−0,23
δ^15^N	0,80	0,03
N	0,79	0,58
DPH	0,76	−0,22
δ^13^C	−0,63	0,42
C/N	−0,66	−0,69
carbohydrate/lignin	−0,95	0,17
carbohydrate/aliphatic	−0,95	0,13

The second component, Cp2, is characterised by positive moderate loadings of N and δ^13^C and a moderate negative loading of the C/N ratio. This indicates that a significant part of the change (close to half of the variance) in the C/N ratio, and smaller proportions of the variation in N and δ^13^C, does not depend on long-term peat decomposition. The opposite loading of the C/N ratio and N indicates that the increases in C/N reflected by this component are accompanied by relatively low N contents (i.e., opposite to the conditions reflected by Cp1). The Cp2 profile is dominated by the large negative scores during 7740-6667/6360 cal yr BP as well as during the HPAE with smaller changes occurring in the rest of the core (Fig. 2d). This suggests that the peat layers in the HPAE have higher C/N ratios than those expected from long-term peat decomposition as represented by Cp1. C/N ratios, after detrending from the Cp1 control, are highly correlated to Cp2 scores (*n* = 105; r = −0.91; *P* < 0.001) and the detrended N contents (*n* = 105; r = −0.84; *P* < 0.001). Thus, Cp1 and Cp2 point to a specific signal of the OM during the HPAE characterized by  well preserved peat that is  relatively low in N contents and has higher C/N ratios. In fact, for samples with negative Cp2 scores (i.e. the earlier excursion and the HPAE) the carbohydrate/lignin ratio is highly correlated (*n* = 27; r = 0.83; *P* < 0.001) to the C/N ratio, indicative of a higher abundance of easily degraded compounds (polysaccharides).

Molecular data for peat layers spanning the period from 6000 to 4000 cal yr BP confirm that the HPAE OM is enriched in labile components (polysaccharide products and 4-isopropenylphenol from sphagnum acid)^33^, while the sections just prior to and after the HPAE are enriched in more recalcitrant lignin and especially aliphatic products, respectively, that occur in more-decomposed OM^26^. This is expressed by negative correlations between DPH and the sum of polysaccharide products (*n* = 20; r = −0.53; *P* < 0.05) and 4-isopropenylphenol (r = −0.87; *P* < 0.001), and positive correlations with sum of *n*-alkanes and *n*-alkenes (r = 0.53; *P* < 0.05) and lignin phenols (r = 0.70; *P* < 0.001). Parameters related to potential effects of vegetation change on OM composition^33^ showed no excursions during the HPAE, confirming that decomposition is the main control on the OM quality of the HPAE. Indeed, the Cp1 scores of these selected samples are correlated with the proportions of aliphatic compounds (*n*-alkanes, *n*-alkenes and *n*-alkanoic acids; *n* = 20; r = 0.84; *P* < 0.001) and negatively associated with carbohydrate products (*n* = 20; r = −0.88; *P* < 0.001), showing that the Py-GC-MS data are in agreement with the other organic proxies and, in particular, for the HPAE. Besides selective preservation of relatively recalcitrant lignin and aliphatic OM during high decomposition periods, there is a positive correlation between Cp1 and pyrolysis products diketodipyrrole (*n* = 20; r = 0.74; *P* < 0.001) and toluene (*n* = 20; r = 0.80; *P* < 0.001), which is indicative of the formation of some microbial biomass as well. This microbial signal is very low during the HPAE, probably as a result of the larger plant bioproductivity compared to microbial degradation. The fact that the HPAE reflects the accumulation of polysaccharides-rich, poorly decomposed plant remains, and not a species change, is also seen in macrofossil evidence from previous studies^16,17^ as well as picking of specimens for dating purposes that reveals the dominance of *Sphagnum* (*fuscum)* species during this interval.

Humification and dust deposition data from Store Mosse suggest that the climate during the HPAE was relatively wet^18^. This is corroborated by regional lake-level records^34,35^ and the generally wet and cold conditions described in western Europe at this time^36^, possibly linked to the abrupt termination of Bond Event 4^37^ (Fig. 2e). These conditions would have led to a higher water table, partly maintained by the vegetation and its associated hydrological conductivity^1^, and increased gross primary productivity in this bryophyte dominated system^38^. Despite Store Mosse and Draftinge Mosse being typical of bogs however, the magnitude of the HPAE is far above that normally observed^22^. As such, we hypothesize that another driver of peat accumulation beyond hydrological changes must have been active at this time.

**Figure 3:**
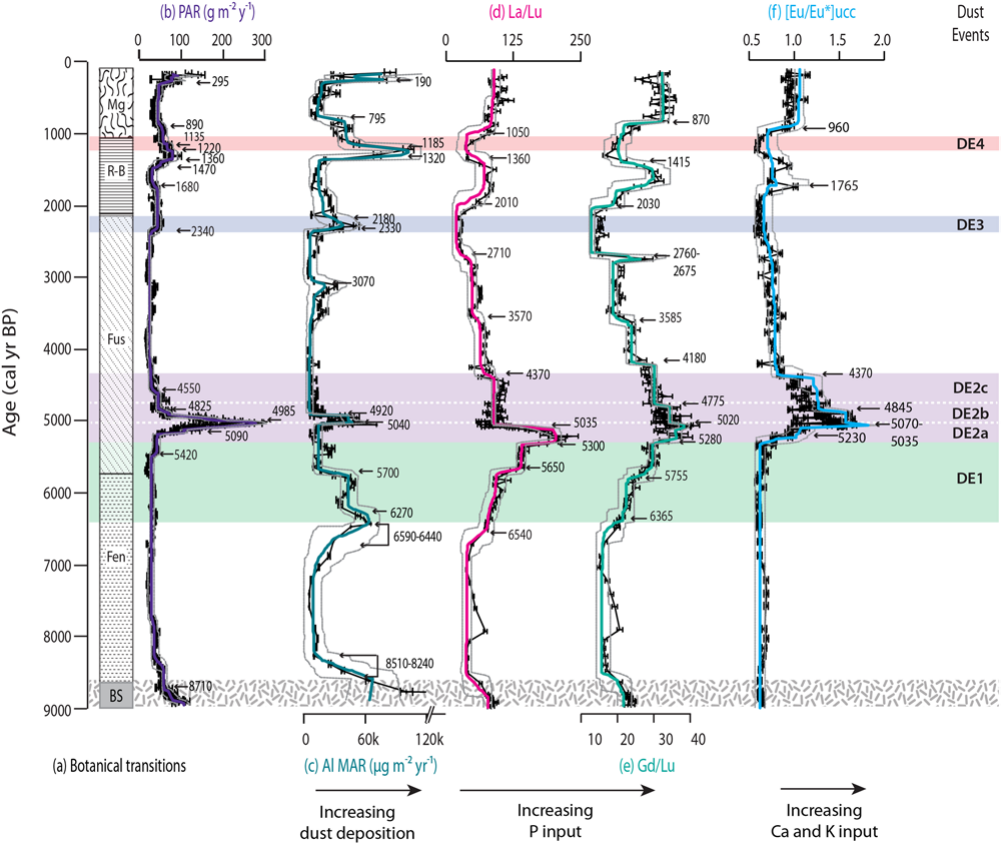
Peat Accumulation Rates (PAR), Al Mass Accumulation Rates (Al MAR) and mineralogical proxies. The HPAE between 5420 and 4550 cal yr BP (**b**) overlaps in time with DE2, which, although having somewhat higher Al MAR, still represents a period of low dust deposition rates (**c**). Rather, DE2 is characterized by shifts in LREE/HREE (**d**) and MREE/HREE (**e**) fractionation and an increase in the Eu anomaly (**f**). Together this indicates the input of less weathered, phosphate and feldspar minerals. Errors on the PAR and Al MAR are the quartiles (25 to 75% of the data) from Monte Carlo simulations (see Methods). Error bars on the ratios are based on the RSD of the same ratios calculated from triplicate analyses of the LKSD-4 reference material. The solid coloured lines are the expected or mean value of the inferred function over time for each data type and the grey dashed lines are the 95% credible intervals from changepoint modelling.

**Figure 4:**
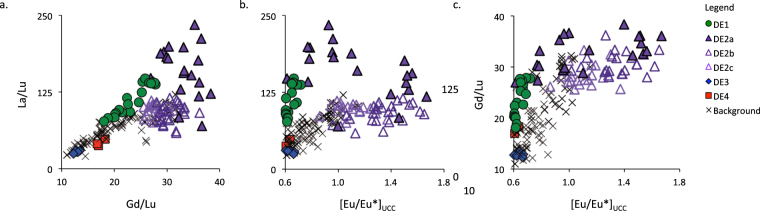
REE fractionation and Eu anomaly. Elemental biplots show that DE2 is enriched in both LREE (La/Lu) and MREE (Gd/Lu), signalling the increased presence of phosphate minerals. [Eu/Eu*]_UCC_, which is controlled by plagioclase feldspar and signals less weathered materials, is increasingly positive during DE2. This combination of minerals would see the addition of both macro- and micronutrients to the nutrient-poor ecosystem of Store Mosse.

Past studies based on the inorganic geochemistry from Store Mosse reveal three periods of increased dust deposition as estimated here using Al MARs (DE1, DE3 and DE4, Fig. 3c), which is representative of that pattern seen for a suite of crustal elements (Sc, Th, La, Y and ∑REE)^18,19^. Based on the relatively high concentrations of crustal elements during DE1, DE3 and DE4, these events have been identified as being more clay-rich in comparison to other parts of the record^19^. During the HPAE there is, however, a distinct change in the quality of the deposited dust. This event can be considered in three phases denoted DE2a, b and c. There is a rapid increase in light (LREE) and middle (MREE) REE enrichments in DE2a as expressed by La/Lu and Gd/Lu, respectively (Fig. 3d,e). LREE enrichments drop sharply in DE2b while MREE enrichment tapers more gradually on into DE2c. The unique character of the LREE and MREE enrichments in DE2 is clear in biplot space (Fig. 4a–c) and is interpreted to signal the presence of the primary phosphate minerals apatite, allanite and/or monazite^19^. The Eu anomaly profile ([Eu/Eu*] _UCC_) closely mirrors that of Gd/Lu during DE2 (Fig. 3f). The Eu anomaly is controlled by plagioclase feldspar (NaAlSi_3_O_8_-CaAlSi_2_O_8_). Studies have also found a negative correlation between Eu anomalies and the chemical index of alteration (CIA) in sedimentary rocks formed from Archean terrains, as well as young deep-sea sediments from a range of tectonic settings. This is due to the fact that high-temperature minerals having a positive Eu anomaly, such as plagioclase feldspar, are prone to weathering, while lower-temperature minerals having a negative Eu anomaly, like potassium feldspar, are less prone. Therefore, as weathering increases the negative anomaly becomes more intense^39^. This does not exclude the presence of alkali feldspars (KAlSi_3_O_8_-NaAlSi_3_O_8_), however. DE2, and DE2a in particular, has been established to be highly felsic (i.e., quartz and feldspar rich) in nature^19^. The input of feldspar is corroborated by the increase in Al MARs during DE2a and b (Fig. 3c). The inorganic geochemistry thus shows that on contrast to DE1, DE3 and DE4, DE2 is characterized by the input of less weathered, phosphate and feldspar minerals.

**Figure 5:**
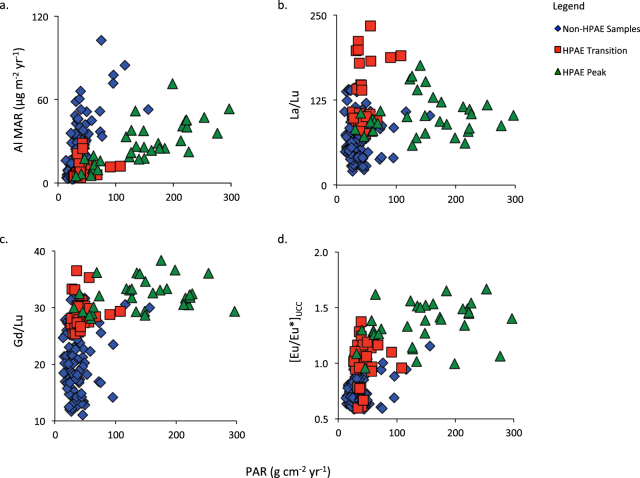
Biplots of Peat Accumulation Rates (PAR) versus Al Mass Accumulation Rates (Al MAR) and mineralogical proxies. Biplots of PAR versus Al MAR show that there is only a weak relationship (*n* = 188, r = 0.12, *P* > 0.05) between these two parameters. Rather, it is the change in mineralogy that is the important control on PAR during the HPAE as indicated by the weaker, although still statistically significant, relationship between PAR and La/Lu (r = 0.21, *P* < 0.05) and the stronger correlation between PAR and Gd/Lu and [Eu/Eu*]_UCC_ (r = 0.46 and r = 0.66, respectively, *P* < 0.001). In these plots the HPAE is separated from the rest of the record (in blue) and divided into the periods of initial increase (5420 to 5090 cal yr BP) and final decrease (4825 to 4550 cal yr BP)(both in red) and the main HPAE peak (5090 to 4825 cal yr BP)(in green).

An influx of phosphate, plagioclase feldspar and overall less-weathered minerals would provide an increased supply of limiting nutrients like P and K as well as micronutrients like Na, Ca and Si, which can boost productivity. The statistically significant correlation observed between PAR and our mineralogical indicators would suggest that this is in fact, what occurs. The correlation  between PAR and Gd/Lu and [Eu/Eu*]_UCC_ (*n* = 188; r = 0.46 and 0.66, respectively; *P* < 0.001) is however stronger than that for PAR and La/Lu (r = 0.21; *P* < 0.05). The importance of the dust quality over the quantity is evidenced by the lower correlation between PAR and Al MAR (r = 0.21, *P* > 0.05) and the greater scatter between these parameters in biplot space (Fig. 5a–d). This boosted productivity is enhanced by the higher water table of the time, which would decrease peat decomposition, further increasing PAR. A similar, although unrelated, HPAE was reported for a peat bog in Japan where CAR increased to >100 g C m^−2^ yr^−1^ over a few centuries. The authors also evoke fertilization via atmospheric deposition to explain the observed accumulation increase but in the form of tephra rather than mineral dust^40^.

Given the coarse nature of the primary minerals (>2 μm) deposited during DE2, we postulate that one or more local or near-regional dust source became active during this time^19^. Store Mosse is nestled at the southern edge of the Southern Swedish Highlands and occupies a broad and shallow low-lying basin (160–170 masl). After deglaciation this area was flooded by an extensive, or a series of interconnected, glacial lake(s), with a potential area of ~800 km^2^. With the eventual drainage of the so-called Lake Fornbolmen, its dry sandy lakebed was eroded by wind, creating a number of sand sheets and dunes in the region, and an ideal substrate for extensive wetland development (Fig. 1a). In addition to these aeolian deposits, erosion by the Scandinavian ice sheet of the granitic and gneissic bedrock of southern Sweden left behind glacial deposits such as tills, sandy remnant lake beds, moraines and eskers^41^. Some of these deposits, as in the case of those located directly to the east and northeast of Store Mosse, are over 50 m thick^42^. A granite-syenite saprolite of higher nutrient content (notably P) and distinct mineralogical composition is also located just 20 km northeast of Store Mosse (the Vaggeryds syenite)^43^. Inputs from these diffuse range of sources could have been activated due to stronger winds associated with the stormier conditions seen 5200–5100 cal yr BP as recorded in southern Sweden^44^, Ireland^45^ and Denmark^46^.

Store Mosse is thought to have reached its lateral extent rapidly^47^. If we assume  that the HPAE is present in equal thickness over the entire bog and that the bog area in the mid-Holocene was the same as present-day, we estimate that 0.5 Mt C was buried during this event. The bogs sitting on the remnant lake bottom of Lake Fornbolmen, including Store Mosse and Draftinge Mosse, currently cover 287 km^2^ (Fig. 1a). Given the similarity in substrate, surroundings and climate, it is likely that there have been similar variations in peat accumulation across the region. Upscaling to reflect this would see an upper estimate of 1.8 Mt C buried over ~1000 years in a relatively small geographical area. During the HPAE dust deposition rates are low with a maximum net dust deposition rate of only 0.88 μg m^−2^ yr^−1^ (estimated using upper continental crust values and Al MARs)^19^. Rather than absolute amount, the mineral composition and character of the dusts deposited at this time appear to have a disproportionately large impact on primary production. While we consider the observed HPAE to be a regional event, created by a unique combination of hydrological shifts, climatic conditions and nutrient-rich local dust sources, the existence of HPAE in peatlands here, and elsewhere, affects our estimation of carbon stocks in northern regions. This work shows that even in the temperate northern latitudes, often overlooked by the dust community, dust dynamics can be an important driver of ecosystem productivity, and thereby, carbon storage and global climate.

## Methods

The data used in this work is available at 10.1594/PANGAEA.887309.

### Sampling

In November 2008 cores were recovered from Store Mosse using a Russian corer (diameter 7.5 cm). Eight overlapping 1 m sections were taken in two adjacent parallel holes. The cores were then aligned using changes in bulk density. Cores were frozen prior to sub-sampling in contiguous 1 cm slices using a stainless steel saw. Samples were then freeze-dried. Approximately a quarter of each sample was saved while the remainder was milled using a plant mill equipped with Teflon vials and agate milling balls.

### Basic Sequence Parameters

Bulk density was calculated for each sample by dividing the dry weight of a sample slice by its estimated volume. PAR was calculated using the bulk density and the accumulation rate derived from the age model (see below). The error on the PAR was estimated by doing a Monte Carlo simulation (n = 100 000) for each sample. For the bulk density measurements the error on radius and thickness measurements was assumed to be ±0.1 cm and ±0.002 g on the weight measurements while the error on the accumulation rate was set at 5%. The distribution was assumed to be uniform for all variables. Humification was determined using a standard procedure^48^. The median absorbance value was taken as the working value although triplicates did not generally vary more than 3%. The absorbance values were corrected for decomposition over time using a first order polynomial regression equation. For stable isotope analysis made each 5 cm the samples combusted with a Carlo Erba NC2500 analyser connected via a split interface to reduce the gas volume to a Thermo Delta V advantage mass spectrometer. From these measurements the reproducibility was calculated to be better than 0.15‰ for δ^13^C and δ^15^N. Carbon and nitrogen values were determined simultaneously when measuring the isotope ratios. The relative error was <1% for both measurements.

### Age Dating

The age model for Store Mosse is based on a total of 19 ^14^C dates. Where possible plant macrofossils were selected for analysis, although in one sample no discernable plant parts were present so bulk peat was sampled. Details of the dated material from Store Mosse are given elsewhere^19^. The age model was built using the CLAM program (v. 2.2)^20^, which includes calibration of the ^14^C dates using IntCal13.14 C calibration curve^49^. The best fit was obtained using a smooth spline and there were no statistical outliers.

### Elemental Concentrations by ICP-MS/ICP-OES

All geochemical sample preparation was performed under clean laboratory conditions using acid-cleaned labware. An initial sampling resolution of 5 cm was used and then areas of greater interest, like the HPAE, were measured at increased resolution for a total of 188 analyses. Multi-element analyses were made on the material remaining after dry ashing at 450 °C overnight. Samples were digested using a MARS-Xpress microwave system and a HNO_3_/HBF_4_ mixture^50^. ICP-AES analyses were made at the Department of Geological Sciences, Stockholm University, Sweden on a Varian Vista AX. ICP-MS analyses were made on a Perkin Elmer Sciex Elan 6000 at the Department of Water Environment Technology, Chalmers University of Technology, Sweden.

Full details of the analytical performance, including results from reference material analyses, are given elsewhere^19^. The analytical performance was assessed through related, although not matrix matched, materials which all have certified/recommended/provisional values for some of the REE including NIST SRM-2711a Montana Soil, Lake Baikal deep-water bottom silt reference material (BIL-1)(Russian Institute of Geochemistry)^51^ and LKSD-4 (Geological Survey of Canada)^52,53^. For the elements used here, namely Al, La, Sm, Gd, Eu and Lu, NIST SRM 2711a showed accuracies of 10% or better for Al, Sm and Eu. Lower accuracies are reported for Gd (28%) and La (35%). The reproducibility was generally <15% with the exception of Al (20%) and Eu (26%). The pattern of elemental recovery and replicate measurements is somewhat similar for BIL-1. Accuracies of 10% or better are calculated for Al, Sm and Eu. Lower accuracies are reported for La (34%), Gd (30%) and Lu (29%). The reproducibility was <15% for La and <21% for Al and Sm. Reproducibilities for the remaining REE were between 24% and 40%. LKSD-4 has accuracies of <12% for Al, Nd, Sm, Eu and Gd but was lower for La (30%). Reproducibilities were <13% for all the REE with the exception of Eu (20%). Slightly higher reproducibilities are observed for Al (21%). Maximum procedural blanks were no greater than 1% of the average peat sample concentrations for the REE with the exception Gd (2%). Slightly higher percentages were observed for Al (8%).

Of the three reference materials the most matrix matched in terms of composition (i.e., granitic shield setting) is LKSD-4. It is also the reference material with the highest organic content, although not nearly at the same levels as the peat measured here. The reported reproducibilities are acceptable for a wet digestion (i.e., <20%). The error on the Al MAR was estimated by doing a Monte Carlo simulation (n = 100 000) for each sample using the generated PAR error and the Al concentration 2 standard deviations (2 SD). The distribution was assumed to be normal for the Al concentrations.

### FTIR-ATR

FTIR-ATR spectra were acquired at 4 cm^−1^ resolution in the region 4000–400 cm^−1^, by averaging 200 scans, using a Gladi-ATR (Pike Technologies) spectrometer at the IR-Raman facility of the RIAIDT of the University of Santiago de Compostela, Spain. Two peat decomposition indices were calculated from each individual spectrum using peak intensities of characteristic bands of carbohydrates (1040 cm^−1^, C-O stretching), lignin (1650 cm^−1^, C=C stretching of aromatic moieties), and lipids (2920 cm^−1^, CH_2_ stretching) (see for example^54^: carbohydrates/lignin (1040/1650 cm^−1^), carbohydrates/lipids (1040/2920 cm^−1^).

### Pyrolysis Gas Chromatography-Mass Spectrometry

Twenty samples were selected from the depth range of 450 to 300 cm (i.e., bracketing the HPAE) and analysed by Py-GC-MS. Samples in quartz tubes were pyrolyzed at 650 °C for 20 seconds using a Pyroprobe 5250. The pyrolysis products were separated on an Agilent 6890 gas chromatograph equipped with a non-polar HP-5MS 5% phenyl, 95% dimethyl-polysiloxane column. The quadrupole mass spectrometer (Agilent 5975) operated in electron impact (70 eV) and scanned in the 50–500 *m*/*z* range. Details can be found in Kaal *et al*.^55^. We denoted the ca. 50 largest peaks in each pyrolysis chromatogram. Relative proportions (%) of the resultant 87 compounds were expressed as the peak area of the dominant ion fragment(s) of a given compound divided by total quantified peak area.

### Principal Component Analysis

Principal components analysis (PCA) was applied to selected properties of the peat OM (total C and N contents, C/N ratio, degree of peat decomposition, and FTIR-decomposition ratios). PCA was carried out on correlation mode, after all variables were standardized (i.e. z-scores) to avoid scaling effects and provide average centred distributions^56^.

### Changepoint Modelling

To quantify the timing (or depth) of discrete changes in the data, a method known as changepoint modelling was used on the individual datasets. A changepoint marks where the running mean of the data changes abruptly. Following the approach described in Gallagher *et al*.^21^, it is possible to infer the location of the changepoints without specifying in advance how many changes there are. It also does not require a priori knowledge of the noise level arising from analytical procedures and/or complexity due to geological processes in the observed values. Here we choose to infer significant jumps in the mean value for a given variable. The variability of the observations about the mean between two changepoints is indicative of the level of noise. The approach uses transdimensional Markov chain Monte Carlo to sample many possible solutions (with different numbers and locations of changepoints and noise estimates), which are either accepted or rejected, based on simple probabilistic rules. The approach is formulated in a Bayesian context, which naturally balances the requirement of fitting the data well with the desire to avoid unjustified complexity in the changepoint structure (i.e., having too many changepoints leading to fitting the data exactly). The output of this method includes probability distributions on the number and location of changepoints, as well as a distribution of the variance of the noise (which is assumed to the variance of a normal distribution).

### Model of Lake Fornbolmen

Differential land uplift following deglaciation has resulted in the redistribution of surface hydrological pathways and lake basins over time. To reconstruct the likely extent of former lakes in the Store Mosse region, we identify closed basins from an isostatically-corrected topography. The isostatic anomaly between the present day and recently deglaciated southern Sweden was extracted from the ICE-6G global palaeotopography model^57^, for 1 kyr timeslices from 13–9 cal. ka. These gridded anomalies were each smoothed and resampled to a cell size of 100 m, and applied to a high-resolution digital terrain model of southern Sweden (© Lantmäteriet). At each timeslice, we used a combination of GIS terrain analysis methods to identify the likely extent of palaeo-lake basins: (i) visual examination of 1 m-interval contours of the corrected topography to identify closed basins and likely spill routes; (ii) automated filling of terrain depressions, either accepting or suppressing local thresholds <0.5 m deep, and using either the 100 m resolution terrain grid or a higher, 20 m resolution surface; and (iii) the same analyses performed on terrain in which the thickness of surficial sediments, derived from the Geological Survey of Sweden (SGU) sediment depth database, was removed from the terrain model for areas where the main sediment unit has been classified by SGU as glaciolacustrine or post-glacial. We find that the greatest area is inundated between 12–10 ka. We note that our analysis does not include the bathymetry of modern lakes (Bolmen and Visöstern) therefore our reconstruction would likely extend with the inclusion of this information.

### Draftinge Mosse

In May 2014 cores were recovered from Draftinge Mosse using a Russian corer (diameter 7.5 cm). Five overlapping 1 m sections were taken in two adjacent parallel holes. The cores were then aligned using changes in bulk density. Cores were frozen prior to sub-sampling in contiguous 1 cm slices using a stainless steel bandsaw. Samples were then freeze-dried. Bulk density was calculated for each sample by dividing the dry weight of a sample slice by its estimated volume. At Draftinge Mosse the age-depth model was obtained using 14 ^14^C dates from Tandem Laboratory, Uppsala University (Ua) and Beta Analytic (Beta) applying the same approach as for Store Mosse. Two outliers were identified but in contrast to the other samples from the fen stage, these measurements were made on bulk sample rather than macrofossils (Table 2).

**Table 2:** Age dating information for Draftinge Mosse.

Sample (cm)	Laboratory no.	Dated Material	14 C yr ± 1 SD	Calibrated Age Range
28	Ua-010650	Bulk peat	554 ± 23	524–561
49	Ua-010654	*Sphagnum* leaves & stems	835 ± 24	594–786
77	Ua-010651	Bulk peat	1259 ± 24	1173–1279
97	Beta-418758	*Sphagnum* stems, seeds	1970 ± 30	1866–1993
117	Beta-418759	*Sphagnum* stems, seeds, insect remains	2390 ± 30	2346–2491
136	Beta-385361	*Sphagnum* leaves, insect remains, charcoal	4070 ± 30	4499–4644
179	Ua-010655	*Sphagnum* leaves & stems	4401 ± 26	4866–5047
219	Ua-010656	*Sphagnum* leaves & stems	4485 ± 25	5211–5278
247	Beta-387826	*Sphagnum* leaves and stems	4690 ± 30	5326–5405
292	Beta-385362	Intact pine cone	4740 ± 30	5510–5580
308	Ua-010652	Bulk peat	5763 ± 26	6492–6639
323	Beta-387827	Charcoal, *Carex*	5180 ± 30	5906–5990
338	Ua-010653	Bulk peat	6932 ± 27	7689–7828
356	Beta-385363	Charcoal, *Carex*	6470 ± 30	7321–7432
389	Beta- 385364	Seeds, insect remains, *Carex*	7510 ± 30	8299–8391
